# The impact of nutritional management on the growth and nutritional status of children on regular hemodialysis

**DOI:** 10.1007/s00467-025-06940-w

**Published:** 2025-10-10

**Authors:** Heba Mostafa Ahmed, Esraa Abdelal Shafey, Rehab Muhammad Abd El kareem, Wesam Ali Ibrahim, Osama Ezzat Botrous

**Affiliations:** 1 Department of Pediatrics, Faculty of Medicine, Beni-Suef University, Beni-Suef, Egypt; 2 Department of Clinical and Chemical Pathology, Faculty of Medicine, Beni-Suef University, Beni-Suef, Egypt; 3 Unite of Metabolism and Nutrition, AboelRish Hospital, Cairo University, Cairo, Egypt

**Keywords:** Nutritional status, Growth, Hemodialysis, Children

## Abstract

**Background:**

Chronic kidney disease (CKD) is a relatively uncommon disease in children. A child's nutritional status indicates how well their body is getting the required nutrients. This research aimed to determine the nutritional state of hemodialysis-treated children with end-stage kidney failure.

**Patients and Methods:**

A case group of 50 children with kidney failure undergoing hemodialysis was compared with a control group of 50 healthy children of the same age. A structured interview questionnaire was used with the children or their mothers, covering nutritional assessment, physical examination, anthropometric measurements, and lab tests. Dietary intake was evaluated through 24-hour recalls, and each case’s dietary consumption was analyzed, followed by nutritional education. Children were reassessed after a 6-month follow-up.

**Results:**

Most children with kidney failure initially had height and weight below the 5th percentile, with Mid Upper Arm Circumference (MUAC) below -3 SD. Dietary recall initially revealed low caloric intake (p = 0.046), high sodium (p = 0.06), high potassium (p = 0.01), high phosphorus (p < 0.01), and low calcium (p < 0.009) compared to controls. By study’s end, MUAC and weight percentiles improved (p = 0.045 and 0.039). Lab results showed significant decreases in urea (p = 0.005), creatinine (p < 0.001), sodium (p < 0.001), potassium (p = 0.001), uric acid (p < 0.001), phosphorus (p = 0.04), and parathormone (p = 0.03) from baseline.

**Conclusion:**

Our study suggests that appropriately targeted nutrition education in children with CKD can improve weight centiles and MUAC and deranged renal biochemistry (including low serum albumin and raised serum potassium).

**Graphical abstract:**

**Figure:**
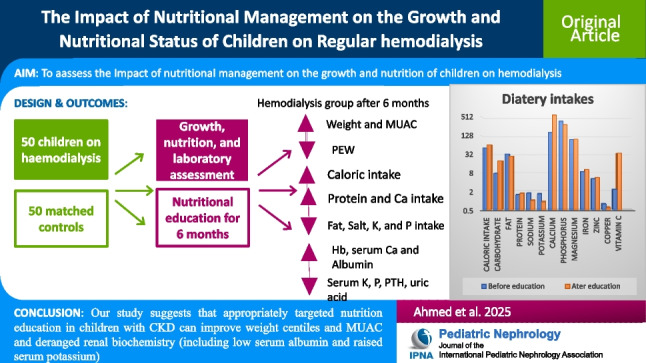
A higher resolution version of the Graphical abstract is available as Supplementary information

**Supplementary Information:**

The online version contains supplementary material available at 10.1007/s00467-025-06940-w.

## Introduction

Chronic kidney disease (CKD) is one of the significant public health concerns. Since CKD is often asymptomatic, particularly in the early stages, epidemiologic statistics may understate the actual frequency and prevalence of CKD. Despite the rising incidence in some countries, CKD is a relatively uncommon disease in children [1].

For CKD patients on dialysis, malnutrition is a frequent complication. The condition is typically seen in protein-energy malnutrition (PEM) or protein-energy wasting (PEW) cases. When PEW ensues, protein reserves (muscle mass) and energy stores (fat mass) are significantly impacted. Data about the prevalence of PEW in children with CKD is limited. According to current data, 15–75% of dialysis patients have PEM in adults [2]. In children with CKD, malnutrition can lead to stunted growth, worsening uremic symptoms, protein-energy loss, and an increase in mortality. In addition, the global epidemic of obesity has substantial impacts on the CKD community [3].

There is an evident paucity of professional dietetic input and variability in the dietary practices for children with CKD, even at tertiary pediatric nephrology clinics [4]. Malnutrition is one of the broadly recognized causes of poor growth in children with CKD, with likely inadequate adherence to the standard of care, which now specifies 0.8–1.05 g/kg of protein per day according to height-age of children aged 2—18 years, plus an allowance of 0.1 g/kg/day for patients on hemodialysis [5], considering that increased levels of uremic toxins in children with dialysis inhibit the sense of hunger, causing less protein intake.

Therefore, this study assessed the nutritional status of children with kidney failure undergoing hemodialysis using anthropometric measurements, dietary intake, and biochemical parameters.

## Methods

### Study design and settings

This case–control study was performed on 100 children from May 2022 to May 2024. The participants were divided into two groups: group 1 included 50 children (< 18 years) with kidney failure on regular hemodialysis from both sexes, whereas group 2 included 50 healthy, age-matched children as a control group. We excluded patients with other chronic diseases or infections, chronic or recurrent diarrhea, immunosuppression, acute kidney injury, kidney transplant, recurrent UTI, recurrent infection, recurrent vomiting, and parental refusal of consent from this study. Subsequently, we conducted an interventional study on our cases by applying a nutritional education program over a 6-month follow-up period.

Before commencing the study, ethical approval was obtained from the local ethical committee, and written informed consent was secured from all included children.

### Data collection

All children were subjected to:

#### Full history taking

Which included present history (age, sex, residence, order of child among other siblings, and duration of hemodialysis), family history (consanguinity, medical conditions in other siblings), natal history, prenatal history (gestational age, mode of delivery, birth weight, and NICU admission), developmental history, and past history, including an initial account of recurrent admission, recurrent UTI, recurrent vomiting, and recurrent infection.

#### Full clinical examination

Anthropometric measurements (weight, height, and mid-upper arm circumference; MUAC) were plotted on growth charts for age and sex. Also, vital signs (such as body temperature, pulse rate, and blood pressure) were recorded. Data was collected again at the end of the study period and interpreted again on growth charts.

Height was measured using a wall-mounted stadiometer. Ideally, the same well-trained person took the measurement at each assessment, ensuring that five body points touched the wall (head, shoulders, buttocks, calf muscles, and ankles). The head was kept in the Frankfurt position. Serial measures of height were used. Weight was measured when the child was determined to be at their euvolemic, or “dry” weight, based on clinical status. MUAC was measured by bending the left arm and marking the olecranon process and acromion with a pen. Marking the midpoint between these two marks. With the arm hanging straight down, wrapping a MUAC tape around the arm at the midpoint mark. CDC reference growth charts were used to assess anthropometric data.

#### Nutritional assessment

##### Dietary recall

A complete dietary history was taken by skilled dietitians using dietary habits recall for three successive days to assess dietary intake. Dietary data was then used to estimate the recommended daily intake of calories, macronutrients (protein, carbs, and fat), vitamins, and minerals. The reference ranges were according to KDOQI, 2009 [6]. For a comprehensive assessment, it is advised to undertake three 24-h recalls, one on a weekend and two throughout the week. For children with CKD, dietary reference intake (Recommended dietary allowance and adequate intake, recommended allowance of copper, vitamin C, zinc, sodium, and potassium, and recommended maximum oral and/or enteral phosphorus intake) was compared to the recommended intake according to KDOQI, 2009 [6].

##### Nutritional Education

All cases were subjected to nutritional education, and each patient had an individualized dietary regimen determined according to their condition and guided by the blood pressure and initial laboratory results for each patient. For all cases, the regimen should consider the following: tolerated upper intake level (UL), acceptable intake (AI), recommended dietary allowance (RDA), and estimated average requirement (EAR) according to height and age.

Individualized nutritional education provides patients with written and spoken dietary information and advice on maintaining a healthy and balanced diet. Furthermore, the significance of diligently following the therapy regimen was outlined. In addition, written examples of sample diets tailored to different age groups were provided. Nutritional monitoring and coaching were undertaken weekly for six months by the pediatric dietitian for all cases.

Follow-up laboratory results were collected after the study period, and nutritional education was provided for the cases. The drug history of cases was considered in this study, and cases were asked about drug intake at the start and the end of the study, noting any change in doses or treatment cessation. Medications, iron, erythropoietin, Ca chelators, vitamin D, and anti-hypertensive drugs were reported. Analyzing dietary recall data was done using Egyptian tables of food composition.

#### Investigations

All children were subjected to laboratory investigations; the requested labs are shown in Table 1. Urea, creatinine, Na, K, Ca, P, and alkaline phosphatase were collected pre-session and mid-week session of dialysis. In contrast, the complete blood picture and serum albumin samples were collected post-dialysis. Laboratory reference ranges were according to KDOQI, 2009 [6]. Around 3 mL of blood was withdrawn from the controls and each patient, and was transferred into plain tubes before the dialysis session and allowed to clot. After centrifugation of the clotted blood, the serum was separated and transferred into aliquots, which were to be stored at −20 °C until measurement. This sample assessed urea, creatinine, Na, K, Ca, P, alkaline phosphatase, albumin, cholesterol, serum iron, uric acid, ferritin, and parathyroid hormone (PTH) levels. Patients were instructed to fast for 8 h before sample collection for serum cholesterol.

### Statistical analysis

The research used Microsoft Excel for data entry, coding, and analysis. After that, the data were loaded into SPSS version 20.0 for analysis. The Chi-square test was used to look for differences between the groups, and the qualitative data were presented as numbers and percentages. The Mann–Whitney U test was used for quantitative data (MEDIAN—IQR). Wilcoxon Signed Rank test was used for comparative analysis between baseline and post-intervention values, and the significance level was set at p-values < 0.05.

## Results

The two study groups were matched for age and sex (M/F, 25/25, and 24/26). Comparisons between the two groups regarding the baseline clinical, nutritional, and laboratory data are shown in Table 1.

**Table 1:** Comparison between cases and controls as regards baseline anthropometric measurements and dietary intakes

Median (IQR)	Cases	Controls	P-Value
*Anthropometric measurements*			
Age (years)	12.00 (7.87–13.6)	11.00 (6–12)	0.09
Gestational age (weeks)	38.00 (36–40)	38.00 (37–39)	0.69
Birth weight (kg)	3.00 (2.5–3)	3.20 (2.8–3.7)	0.57
Weight (kg)	20.50 (16.3−28.3)	30.00 (20−37)	0.007
Weight percentile	0.09 (0–2.6)	75.00 (37–90)	< 0.001
Height (cm)	118.50 (104.5–135)	140.00 (112−141)	0.032
Height percentiles	0.03 (0–0.5)	50.00 (32−75)	< 0.001
BMI (kg/m^2^)	14.81 (14.3–17.4)	17.52 (14.9–20.5)	< 0.001
Mid upper arm circumference (cm)	17.50 (16−20)	21.50 (19−23)	< 0.001
*Dietary intakes*			
Caloric intake (Kcal/kg/day)	52.50 (41–66)	55.00 (49–65)	0.046
Carbohydrate (g/kg/day)	7.82 (5.7–9.7)	7.80 (5.3–12)	0.285
% of caloric intake	49.00 (45–57)	59.00 (49–73)	0.046
Fat (g/day)	33.45 (27.35–54.65)	66.20 (39–77)	< 0.001
% of caloric intake	37.29 (29.6–41)	31.80 (23−35)	0.023
Protein (g/kg/day)	1.61 (1.07–2.25)	2.01 (1.5−2.24)	0.02
Sodium (mg/day)	1817 (1575–2478)	2190 (1778−2760)	0.066
Potassium (mmol/kg/day)	1.72 (1.12–2.53)	1.25 (0.92–1.65)	0.01
Calcium (mg/day)	165 (147–205)	222 (121.75–304.50)	0.009
Phosphorus (mg/day)	386 (342−505)	280 (240–361)	< 0.001
Magnesium (mg/day)	98 (64.5–187.2)	90 (60−129)	0.005
Iron (mg/day)	8.81(7.94–10.79)	9.25 (7.44–10.98))	0.002
Zinc (mg/day)	5.23(4.29–6.47)	6.31 (4.2–7.78)	0.029
Copper (mg/day)	0.76 (0.52–1.11)	1.11 (0.74–1.3)	0.029
Vitamin C (mg/day)	2.34 (1–11)	1.93 (1–10)	0.555
*Laboratory data*			
Hemoglobin (g/dl)	10.00 (7.80–12.5)	11.50 (10.5–12)	0.001
Mean corpuscular volume	81.00 (77–84)	78.60 (75−80)	0.07
Leucocyte count/mm^3^ × 10^3^	6.50 (4–7.4)	6.90 (6.3−8.70)	0.004
Platelets/mm^3^ × 10^3^	262.00 (223–329)	344.00 (307–455)	< 0.001
Urea (mg/dl)	120.00 (114–140)	24.00 (20−27)	< 0.001
Creatinine (mg/dl)	7.00 (5.8–9.4)	0.50 (0.50–0.62)	< 0.001
Sodium (mEq/L)	140.00 (136–143)	140.00 (135−144)	0.581
Potassium (mEq/L)	8.22 (6.8–9.2)	4.40 (4–4.8)	< 0.001
Albumin (g/dl)	3.50 (3.3–4)	4.10 (3.9–4.3)	< 0.001
Cholesterol (mg/dl)	176.00 (138–273)	145.00 (129–167)	0.015
Uric acid (mg/dl)	7.10 (5.2–8.2)	4.00 (3.2–5)	< 0.001
Iron (mg/dl)	64.00 (44–69)	133.00 (100–156)	< 0.001
Ferritin (mg/dl)	259.50 (193–334)	150.00 (108−201)	0.026
Calcium (mg/dl)	8.91 (7.9–10)	9.90 (9.5–10)	< 0.001
Phosphorus (mg/dl)	5.70 (3.8–6.7)	4.50 (3.9−5)	0.824
Alkaline phosphatase (u/l)	353.00 (113–687)	158.00 (118−180)	< 0.001
Parathyroid hormone (pg/ml)	556.00 (62–1025)	60.21 (25–72)	< 0.001

### Baseline clinical data and anthropometric measurements of the studied participants.

The median age of patients was 12 years (7.87–13.6), with a median weight of 20.5 kg and height of 118.5 cm. The median weight percentile was 0.09, height percentile 0.03, and MUAC 17.5 cm. The etiology of CKD was CAKUT in 22 (44%), FSGS in 16 (32%), cystic kidney diseases in 8 (16%), other causes in 2 patients (4%), and unknown etiology in 2 patients (4%).

Comparison between cases and controls revealed a significantly lower weight (p-value 0.007), height (p-value 0.032), weight percentile (p-value < 0.001), height percentile (p-value < 0.001), and MUAC (p-value < 0.001).

### Baseline dietary intake among the studied participants

The caloric intake and the caloric intake percentage of expected requirements for height-age were significantly lower in cases compared to the control group (p-value 0.046). No significant difference was observed between cases and controls regarding carbohydrate intake (p-value 0.285); however, fat and protein intake were significantly lower in cases than in controls (p-value < 0.001 and 0.02, respectively).

In terms of minerals, sodium intake was significantly lower (p-value 0.066), as was calcium (p-value 0.009), iron (p-value 0.002), zinc (p-value 0.029), and copper (p-value 0.029) in cases than controls, respectively. On the other hand, the intake of K (p-value0.01), P (p-value < 0.001), and Mg (p-value 0.005) was significantly higher in cases compared to controls. Vitamin C intake showed no significant differences between cases and controls. 30% of the patients had low protein intake compared to that recommended for height-age, while 70% had high intake compared to that recommended for height-age the median percentage of expected requirement for height-age was 146% (88–210%). The median intake, expressed as a percentage of an expected requirement for height-age, was 142% (120–178%) for Na intake, 172% (114–251%) for K, 50% (25–70%) for Ca, and 75% (41–98%) for P.

### Laboratory data results among the studied participants

At baseline, hypernatremia was reported in 8 (16%) patients, hyperkalemia in 38 (76%), hypoalbuminemia in 15 (30%), hypocalcemia in 10 (20%), hyperphosphatemia in 11 (22%), hyperuricemia in 24 (48%), and iron deficiency in 16 (32%). The median Hb level was significantly lower in cases than in controls (p-value 0.001). Also, the total leucocyte counts (TLC) and platelet count were significantly lower in cases than in controls (p-value 0.004 and < 0.001, respectively).

Urea and creatinine levels were significantly higher among cases than controls (p-value < 0.001). While serum Na level showed no significant difference between cases and controls, the serum K level was significantly higher among cases than controls (p-value < 0.001). Also, the serum cholesterol, uric acid, ferritin, phosphorus, alkaline phosphatase, and PTH were significantly lower in cases than controls (p-value < 0.05). On the other hand, serum albumin, iron, and calcium were significantly lower in cases than in controls (p-value < 0.05).

Comparisons between the clinical, nutritional, and laboratory data of the patient group before and after nutritional management are shown in Table 2.

**Table 2:** Comparison between cases before and after the study

Median (IQR)	Before	After	P-Value
*Anthropometric measurements*			
Weight (kg)	20.50 (16.3−28.3)	23.50 (19–31)	0.067
Weight percentile	0.09 (0–2.6)	0.54 (0.07–5.8)	0.039
Height (cm)	118.50 (104.5–135)	125.00 (109–140)	0.213
Height percentiles	0.03 (0–0.5)	0.11 (0–0.7)	0.139
BMI (kg/m^2^)	14.81 (14.3–17.4)	15.23 (14.6–17.8)	0.57
MUAC (cm)	17.50 (16−20)	20.00 (16.7−22)	0.045
*Dietary intakes*			
Caloric intake (Kcal/kg/day)	52.50 (41–66)	65.79 (54–103)	0.001
Carbohydrate (g/kg/day)	7.82 (5.7–9.7)	20.00 (15–30)	0.001
Fat (g/day)	33.45 (27.35–54.65)	28.34 (25–40)	0.03
Protein (g/kg/day)	1.61 (1.07–2.25)	1.80 (1.04−2.05)	0.043
Sodium (mg/day)	1817 (1575–2478)	1044 (467–1692)	0.001
Potassium (mmol/kg/day)	1.72 (1.12–2.53)	0.94 (0.9−1)	0.001
Calcium (mg/day)	165.33 (147–205)	600.41 (510−700)	0.001
Phosphorus (mg/day)	386.00 (342−505)	300.24 (280–350)	0.001
Magnesium (mg/day)	98.43 (64.5–187.2)	100.27 (75–120)	0.002
Iron (mg/day)	8.81(7.94–10.79)	10.45 (10–12)	0.357
Zinc (mg/day)	5.20 (4.29–6.47)	5.70 (5.2–7.3)	0.261
Copper (mg/day)	0.76 (0.52–1.11)	0.59 (0.52–0.78)	< 0.001
Vitamin C (mg/day)	2.31 (1–11)	35.43 (25–45)	0.001
*Laboratory data*			
Hemoglobin (g/dl)	10.00 (7.80–12.5)	10.00 (9.4−13.5)	0.125
Mean corpuscular volume	81.00 (77–84)	81.00 (78–85)	0.171
Leucocyte’s count/mm^3^ × 10^3^	6.50 (4–7.4)	5.70 (4.6–6.1)	0.047
Platelets/mm^3^ × 10^3^	262.00 (223–329)	220.00 (171–230)	0.001
Urea (mg/dl)	120.00 (114–140)	95.00 (77–131)	0.005
Creatinine (mg/dl)	7.00 (5.8–9.4)	4.80 (4.6−6.1)	< 0.001
Sodium (mEq/L)	140.00 (136–143)	136.00 (134−139)	< 0.001
Potassium (mEq/L)	8.21 (6.8–9.2)	6.20 (5.5–7.2)	0.001
Albumin (g/dl)	3.50 (3.3–4)	4.00 (3.6−4.4)	0.002
Cholesterol (mg/dl)	176.00 (138–273)	156.00 (141–175)	0.02
Uric acid (mg/dl)	7.10 (5.2–8.2)	6.00 (5.2–6.6)	< 0.001
Iron (mg/dl)	64.00 (44–69)	55.00 (42–87)	0.159
Ferritin (mg/dl)	259.50 (193–334)	173.00 (134–305)	0.066
Calcium (mg/dl)	8.92 (7.9–10)	9.83 (9.1−10.8)	0.036
Phosphorus (mg/dl)	5.70 (3.8–6.7)	4.50 (3.9–5.4)	0.041
Alkaline phosphatase (u/l)	353.00 (113–687)	370.00 (326–470)	0.068
Parathyroid hormone (pg/ml)	556.00 (62–1025)	492.00 (120−656)	0.034

### Anthropometric measurements at the end of the study

At the end of the study, there was a significant increase in the weight percentile (p-value 0.039) and the MUAC (p-value 0.045). Although the mean weight, height, and height percentile increased at the end of the study duration, no statistically significant differences could be noted. The body mass index (BMI) was comparable at baseline and after 6 months.

### Dietary intake at the end of the study

At the end of the study, there was a significant increase in total caloric intake (p-value 0.001), carbohydrate intake (p-value 0.001), protein intake (p-value 0.043), calcium intake (p-value 0.001), magnesium intake (p-value 0.002), and vitamin C (p-value 0.001). However, there was a significant decrease in the level of fat intake (p-value 0.03), Na intake (p-value 0.001), K intake (p-value 0.001), phosphorus intake (p-value 0.001), and copper intake (p-value < 0.001). There were no significant differences between the intake of iron and zinc before and after the study (p-value 0.357 and 0.261, respectively). At the end of the study, adequate protein intake was reported in 40% of patients. However, intake was still high at 60%, with about a 30% reduction rate compared to baseline. The median percentage of expected requirement for height-age was 140% (100–187%) for protein intake, 90% (62–98%) for Na intake, 56% (25–65%) for K, 100% (50–175%) for Ca, and 36% (20–75%) for P.

### Laboratory data at the end of the study

At the end of the study, there was a significant increase in intake of total calories (p-vlue 0.001), carbohydrate (p-value 0.001), protein (p-value 0.043), calcium (p-value 0.001), magnesium (p-value 0.002), and vitamin C (p-value 0.001). However, there was a significant decrease in intakes of fat (p-value 0.03), Na (p-value 0.001), K (p-value 0.001), phosphorus (p-value 0.001), and copper (p-value < 0.001).

## Discussion

As children with CKD age and their disease progresses, maintaining optimal nutrition becomes more challenging, necessitating frequent updates to the care plan. A kidney dietitian is vital in meeting calorie, protein, and electrolyte needs within dietary restrictions [7]. This study compared 50 children with kidney failure on hemodialysis and 50 healthy age-matched controls, assessing growth and nutrition through anthropometric and serum measures (albumin, uric acid, cholesterol) after six months of dietary management.

In this study, 56% (28 of 50) of CKD cases were female. Glomerular causes were the primary etiology in 14 males and 24 females (male-to-female ratio 1:1.7). At the same time, CAKUT was more common in males (male-to-female ratio 2:1). This aligns with Denburg et al., who found immune and glomerular conditions to be more prevalent in females, and urological causes like PUV more common in males [8]. The baseline median weight was 20.5 kg, with cases falling below the 3rd percentile on growth curves and significantly lower in body weight and weight percentiles than controls (p-value 0.007 and < 0.001). Previous studies by Abraham et al. [9], Gupta et al. [10], and Zahed et al. [11] also reported growth failure, particularly low weight, in children with CKD.

By the end of the study, patients’ weight increased slightly but not significantly (p-value 0.06), though weight percentiles showed a significant rise (p-value 0.03). Marlais et al. [12] reported that children with CKD on tube feeds improved from −2.33 SD to −1.45 SD in weight after one year and −1.39 SD after two years (p-value < 0.001). Height was also impacted, with a median of 118 cm at entry, significantly lower than controls (p-value 0.03) and below the 3rd percentile on growth charts compared to the 50th percentile for controls (p-value < 0.001). This aligns with Gupta et al. [10] and Silva et al. [13], who highlighted growth retardation in children with CKD, with severe short stature found in 36.6% of pre-dialysis and 47% of dialysis children.

About one-third of children with moderate to severe CKD had heights below the third percentile, meaning they were shorter than 97% of their peers; Tonshoff et al. observed similar findings. Growth delays in children with CKD can result from factors such as age at disease onset, primary kidney disease, PEM, metabolic disorders, kidney osteodystrophy, anemia, and urinary electrolyte imbalances [3, 14]. Growth stunting is more likely to occur if CKD begins in infancy, worsening in advanced CKD, with severity inversely related to weight and height [10].

In our study, height measurements and percentiles improved by the end but were not statistically significant. In contrast, Norman et al. found a significant height SDS increase in 63% of children with mild, 47% with moderate, and 69% with severe chronic kidney insufficiency, particularly in those with severe CRI over two years. This difference may stem from our shorter study duration and inclusion of only pre-dialysis children in their study [15]. Improvements in caloric and protein intake, electrolyte balance, and metabolic bone health through dietary management contributed to weight and height gains by the study's end.

MUAC is helpful in assessing nutritional status and growth in patients with CKD. Iyengar and Mak [16] found MUAC highly sensitive (92%) in identifying non-malnourished children and 99% specific for severe malnutrition. Abraham et al. [9] observed that 41% of patients with CKD had reduced MUAC, suggesting muscle wasting occurs before being considered underweight. In our study, the median MUAC was significantly lower in cases than in controls (p-value < 0.001) but increased to a median of 20 cm (z-score −1.9) by the end (p-value0.04). Improved nutrition and education help enhance muscle bulk without raising urea and phosphorus levels. CKD commonly causes PEW, characterized by four primary components: biochemical criteria, such as low serum albumin or low cholesterol, reduced body mass, reduced muscle mass, and decreased body protein [9]. Despite this, serum albumin is commonly used as a biomarker of nutritional status. In patients with CKD, serum albumin lacks sensitivity and specificity as it is influenced by non-nutritional factors, such as fluid overload in dialysis patients, protein loss in urine and dialysate, inflammation, and infection [17] Abraham et al. [9] noted that PEW affects 40% of children with CKD, with MUAC serving as a marker for this type of malnutrition. Muscle loss in CKD results from decreased muscle protein synthesis and increased breakdown, exacerbated by insulin resistance and waste accumulation in kidney failure. Without sufficient dietary protein, CKD patients face heightened protein and amino acid breakdown, leading to lean mass loss and negative nitrogen balance, especially during protein restriction [18, 19].

In our study, 34% of cases had PEW at baseline, which improved to 8% by the end of the study (p = 0.04) due to increased caloric and protein intake, leading to higher serum albumin levels and muscle bulk. The median baseline caloric intake was 52 kcal/kg, below average for age, with 48% of children showing caloric deficiency. This improved during the study to a median of 64 kcal/kg (p-vlaue 0.001). Reduced intake in children with CKD may result from anorexia, chronic illness, fatigue, hormonal imbalances, metabolic issues, inflammation, and dialysis-related factors [18].

Our study found imbalanced macronutrient intake at baseline, with 68% of patients exceeding fat recommendations and 36% falling short on carbohydrates, leading to higher cholesterol levels than controls. After nutritional education and dietary intervention, patients met dietary recommendations for fats and carbohydrates, and cholesterol levels decreased significantly. High fat intake may stem from attempts to replace protein with fat, mistakenly thought to aid growth. CKD-associated lipid issues, like hypertriglyceridemia and low HDL-C, heighten CVD risk [20]. Reducing fat intake can help manage lipid levels, lower cardiovascular risk, and possibly slow CKD progression [21]. Comprehensive lipid guidelines for CKD patients are needed [22].

In our study, initial sodium intake was similar between patients and controls, with 32% of controls and 22% of patients exceeding the recommended daily intake. By the end of the study, patient sodium intake had significantly decreased, accompanied by reductions in systolic and diastolic blood pressure and median serum sodium levels. Similarly, a nationwide study by Garriguet et al. found high sodium intake among healthy children, with 77% of 1- to 3-year-olds consuming 1,918 mg/day, exceeding the 1,500 mg/day limit [23]. Limiting sodium is essential for dialysis patients to control blood pressure and volume [3, 24], as excess sodium can lead to hypertension due to oxidative stress and endothelial dysfunction [25]. Sodium restriction also enhances the effectiveness of anti-hypertensive drugs in patients with CKD [26].

At the beginning of our study, daily potassium intake and serum potassium levels were significantly higher in cases than in controls. By the end of the study, potassium intake and serum levels had decreased significantly. Similarly, a study by El-Sharkawy et al. on 400 hemodialysis patients showed that those on a potassium-rich diet had a higher pre-dialysis serum potassium level (5.691 ± 0.462 mEq/L) compared to patients on an average (4.941 ± 0.376 mEq/L) or low-potassium diet (4.309 ± 0.321 mEq/L), with a significantly higher incidence of hyperkalemia (p < 0.01) in the potassium-rich group [27]. Since kidneys excrete over 90% of potassium, the risk of hyperkalemia increases as glomerular filtration rate (GFR) drops below 10–15 mL/min/1.73 m^2^, or in cases of acidosis, hemolysis, urinary blockage, or use of potassium-sparing medications. High potassium levels can severely impact heart and muscle function, potentially leading to fatal complications like cardiac arrest or respiratory paralysis in advanced CKD. Therefore, potassium intake control is crucial in CKD dietary management [28].

While numerous studies address abnormal potassium levels and related treatments in patients with CKD, little research focuses on the nutritional needs and K⁺ management of children with CKD on dialysis. Findings suggest that a daily intake of 2000–3000 mg (50–75 mmol) or 1–1.3 mmol/kg can help most people with hyperkalemia and CKD maintain normal blood K⁺ levels. Based on adult data, KDOQI recommends a daily K⁺ intake of 1–3 mmol/kg for infants and young children [6]. Few studies explore the impact of potassium intake on CKD outcomes. One study in adults on hemodialysis found that those in the highest potassium intake quartile had a 2.4 times higher mortality risk (95% CI: 1.1–7.5) than those in the lowest quartile [29]. A recent meta-analysis also showed that dietary potassium restriction significantly reduces serum K⁺ levels in CKD patients. However, this finding primarily comes from a single trial using a restrictive liquid diet, which may not be feasible for typical dietary needs [30].

In our study, cases initially had lower daily calcium intake and higher phosphorus intake compared to controls. By the end of the study, Ca intake significantly increased, while P intake decreased. Serum Ca levels, initially lower than controls, increased significantly, while P and parathyroid hormone levels, initially higher, decreased. Consequently, the need for phosphorus chelation also dropped. McAlister et al. [4] found that 76% of children with advanced CKD (stages 4–5D) consumed insufficient calcium, with median dietary Ca intake much lower than age-matched controls. Serum levels of Ca, ionized Ca, PTH, and alkaline phosphatase often did not reflect dietary intake, possibly due to reduced Ca recommendations. Our study participants used higher doses of calcium supplements to meet the daily requirements. Reduced Ca intake resulted from advice to decrease P intake from dairy foods, as the first line of P management involved limiting dairy and avoiding P additives.

Zinc and copper recalls were significantly lower in cases than in controls. Zinc recalls were comparable at the start and the end of the study, while copper recalls were significantly higher at the end of the study. Previous studies have reported that dietary intake less than the DRI has been noted for zinc and copper in children receiving continuous peritoneal dialysis (CPD) [31]. Reduced zinc and copper intake in patients with CKD is related to restricted animal protein intake. Zinc and copper deficiency are attributed to anemia, growth retardation, and bone disease in children on hemodialysis. The guideline recommends dietary intake consisting of at least 100% of the DRI for copper and zinc in children receiving hemodialysis [6].

In our study, serum uric acid was significantly higher in cases than in controls, and it was significantly reduced at the end of the study. Uric acid is excreted mainly by the kidneys. In CKD patients, decreased GFR highly affects its excretion. The significant reduction of uric acid at the end of the study may be due to the lower catabolic state associated with a good nutritional status. Similar to our findings, Xu et al. showed that serum uric acid was categorized as (< 6.5 mg/dL), (6.5–9 mg/dL), or (> 9 mg/dL) in their cross-sectional analysis and found that most of the children between 6.5–9 mg/dL represented the approximate IQR of the distribution. Decreased uric acid level is a critical indicator of decreased gout risk [32].

Additionally, cases had significantly lower baseline iron intake than controls, with no change in iron intake by the end of the study. Hemoglobin and iron levels were lower in cases than controls, while ferritin levels were higher. Hb, iron, and ferritin levels remained consistent within the case group throughout the study. Similarly, Koshy and Geary [33] found that 54.1% of pediatric HD patients had mean annual Hb levels below 11 g/dL, with significantly lower iron levels (p-value < 0.001). Bhagat et al. [34] reported anemia in 34 of 54 children with CKD, and Agarwal [35] found a mean Hb of 9.2 ± 1 g/dL in CKD patients.

Anemia in children with CKD is often due to reduced erythropoietin production from renal damage, with additional contributions from factors like iron deficiency, inadequate diet, poor iron absorption, and blood loss through tests or dialysis. Iron deficiency plays a larger role in early CKD stages, potentially compounded by folate, vitamin C, and B12 deficiencies. Chronic inflammation also exacerbates anemia in CKD, with ferritin levels often being unreliable as markers of iron status due to upregulated ferritin production in inflammation [36]. Over half of CKD patients, especially at advanced stages or on dialysis, experience chronic inflammation, making ferritin levels potentially misleading [35]. In our study, high ferritin levels likely resulted from chronic inflammation, hepcidin upregulation, and parenteral iron intake.

Regarding vitamin C intake, most of our patients showed exceptionally low vitamin C intake before the study; however, this improved dramatically after nutritional education to meet the RDA according to age. It has been documented that patients with CKD, HD, and CPD had low vitamin C levels [37], with low intake (such as reduced fruit intake) and dialysis losses being the causes of low levels observed in dialysis patients [38]. By food alone, 24 out of 30 children received less than the RDA, according to Pereira et al. [39]. As we did not measure vitamin C levels, we could not assess the impact of increased nutritional intake on serum levels.

This study has some limitations; in particular, the number of patients enrolled is small, and the study period was relatively short. Therefore, future studies need to include larger patient groups and monitor them longer to validate our findings.

## Conclusion

Our study suggested that appropriately targeted nutrition education in children with CKD can improve weight centiles, MUAC, and deranged renal biochemistry (including low serum albumin and raised serum potassium).

## Supplementary Information

Below is the link to the electronic supplementary material.

Graphical abstract (PPTX 76 KB)

## Data Availability

All data are provided in the article.
