# Supplementary material for: The impact of nutritional management on the growth and nutritional status of children on regular hemodialysis
Source: Pediatr Nephrol. 2025 Oct 10;41(2):509–18. doi: 10.1007/s00467-025-06940-w (PMC12727846; doi:10.1007/s00467-025-06940-w)
Supplement: Supplementary file 1 — Graphical abstract (PPTX 76 KB) [file 467_2025_6940_MOESM1_ESM.pptx]

## Slide 1
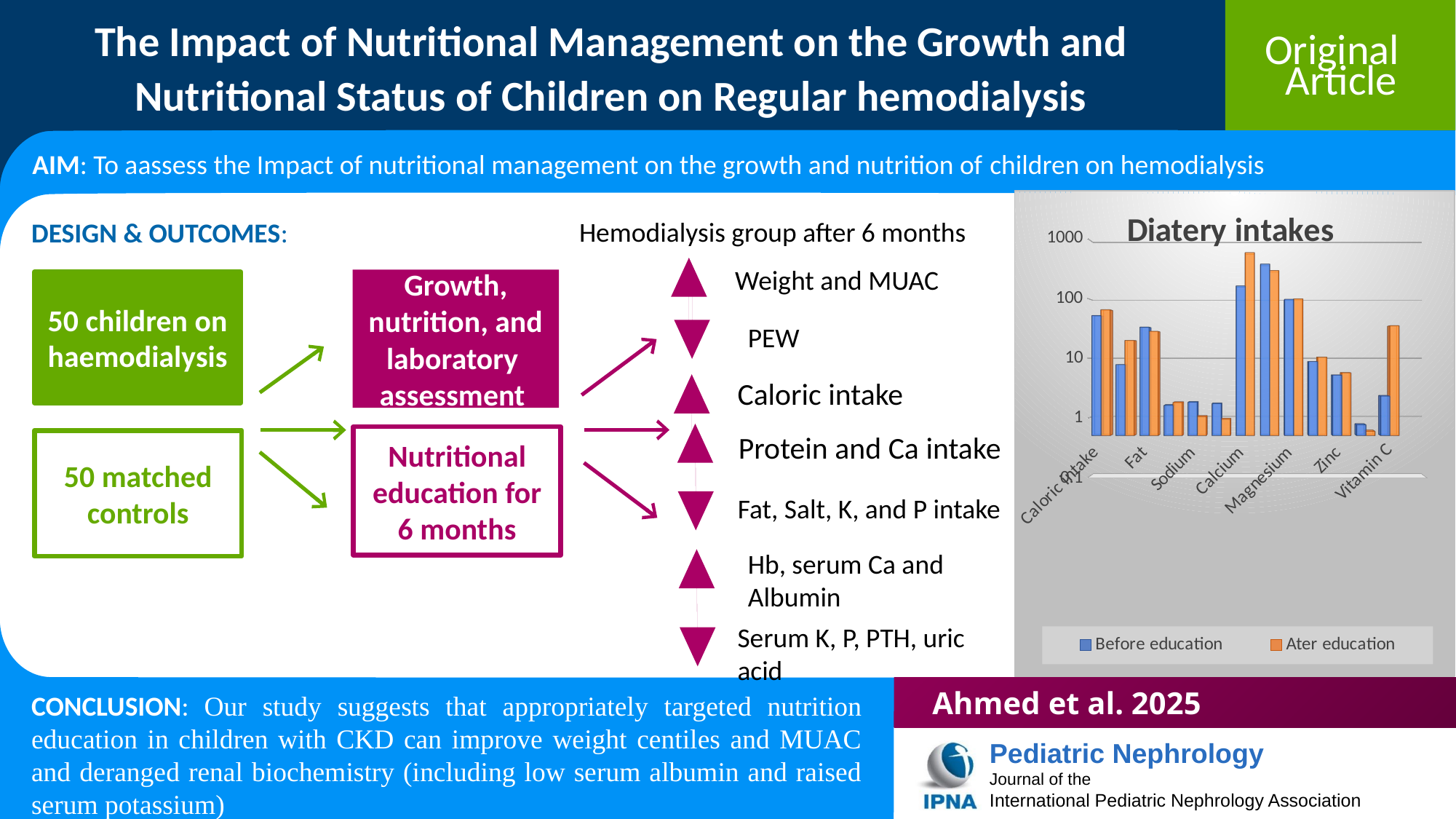

The Impact of Nutritional Management on the Growth and Nutritional Status of Children on Regular hemodialysis
AIM: To aassess the Impact of nutritional management on the growth and nutrition of children on hemodialysis
[unsupported chart]
Hemodialysis group after 6 months
DESIGN & OUTCOMES:
Weight and MUAC
Growth, nutrition, and laboratory assessment
50 children on haemodialysis
PEW
Caloric intake
Protein and Ca intake
Nutritional education for 6 months
50 matched controls
Fat, Salt, K, and P intake
Hb, serum Ca and Albumin
Serum K, P, PTH, uric acid
Ahmed et al. 2025
CONCLUSION: Our study suggests that appropriately targeted nutrition education in children with CKD can improve weight centiles and MUAC and deranged renal biochemistry (including low serum albumin and raised serum potassium)
